# Neuroglial cells: evolving concepts at the West Riding Asylum, England, 1879–1895

**DOI:** 10.1007/s00702-026-03125-z

**Published:** 2026-03-24

**Authors:** Andrew J. Larner, Lazaros C. Triarhou

**Affiliations:** 1 Department of Translational Neuroscience & Stroke, Institute of Neurology, University College London, London, UK; 2 Department of Psychology, Division of Brain, Behavior and Cognition, Aristotelian University Faculty of Philosophy, Thessaloníki, Greece

**Keywords:** History of neuroscience, Neuroglia, Andriezen, Bevan-Lewis, Goodall, Scavenger cells, Spider cells, Golgi stain

## Abstract

In the last quarter of the nineteenth century, three pathologists working in the laboratory of the West Riding Asylum at Wakefield, West Yorkshire, in the north of England, contributed to research on neuroglia: William Bevan-Lewis (1847–1929), Edwin Goodall (1863–1944), and William Lloyd Andriezen (ca.1870–1906). This article examines their respective contributions and notes the evolving trajectory of research from purely descriptive accounts of unitary “spider cells”, possibly endowed with a “scavenger” function as part of the “lymphatic-connective system” (Bevan-Lewis, Goodall), to the subdivision of the class of spider cells with descriptions and illustrations of different types of neuroglial cells courtesy of the Golgi stain (Andriezen). The local interactions of these three researchers are examined as well as the more general impact of their research in the field of neuroglia.

## Introduction

The history of the discovery and characterisation of the non-neuronal cellular elements of the central nervous system in the nineteenth century is generally recognised to have commenced with the concept of *Nervenkitt* (“nerve glue”) advanced by Rudolf Virchow (1821–1902) in the mid-1850s. Virchow adopted his mentor Johannes Müller’s (1801–1858) concept of “connective tissue” (*Bindegewebe*) in his conceptualisation of a “substance which lies between the proper nervous parts” and which he famously named “neuroglia” (Virchow 1858, p. 246, 249–250). The cellular nature of neuroglia became apparent with the drawings of Otto Deiters (1834–1863) illustrating stellate glial cells, most likely astrocytes, published posthumously in 1865 (Deiters and Guillery 2013). As a consequence, these cells became known in some quarters as “Deiters cells”. Moritz Jastrowitz (1839–1912) also examined these cells, noting various morphologies, naming them as *spinnenähnliche Gliazellen* (“spider glial cells”) or *Spinnezellen* (“spider cells”) in the early 1870s (Dalvi 2024). Around the same time, Camillo Golgi (1843–1926) had distinguished different forms of neuroglia, even before the development of his eponymous black reaction (Golgi 1871, 1872), a description later confirmed and expanded using this staining method (Golgi 1885). Further advances later in the nineteenth century included the descriptions and illustrations of Santiago Ramón y Cajal (1852–1934), the discovery by Wilhelm His (1831–1904) of the shared ectodermal origin of neurones and neuroglial cells, and Mihály von Lenhossék’s (1863–1937) coinage of the name “astrocyte” in 1895 (Lenhossék 1895, p. 179–180). Many other researchers also contributed papers on the subject (see supplement to Kettenmann et al. 2025).

In addition to these landmarks, some histories of neuroglia have credited William Lloyd Andriezen (ca.1870–1906) with the earliest description of fibrous and protoplasmic glia, dating from the time when he was working at the West Riding Asylum at Wakefield (WRA), located in West Yorkshire in the north of England. The dedicated pathological laboratory at WRA had been opened in the early 1870s by the then Asylum Superintendent, James Crichton-Browne (1840–1938), and it was here that David Ferrier’s (1843–1928) initial experimental studies of brain physiology which identified the presence of cortical motor centres were undertaken in 1873, findings which helped to establish the concept of cortical localisation. Pathological work had continued at WRA under the Superintendents following Crichton-Browne, namely Herbert Major (1850–1921) between 1876 and 1884 (Larner 2024) and thereafter William Bevan-Lewis (1847–1929).

**Fig. 1:**
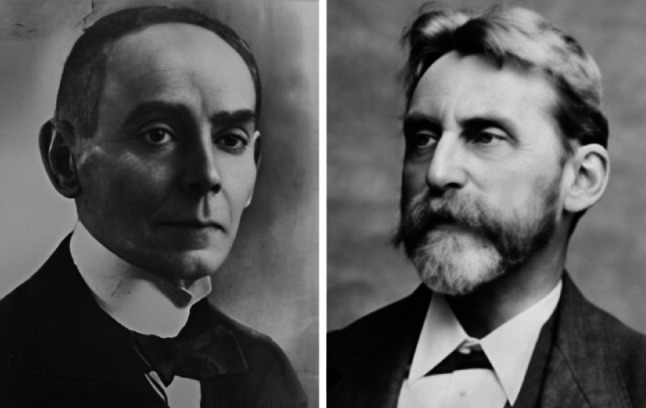
Edwin Goodall, left (adapted from Beech 2011). Bevan-Lewis, right (adapted from a group photo at Wakefield Asylum, c. 1900, from Hoole 2013)

It transpires that Andriezen was not the only, nor indeed the first, pathologist to examine the question of neuroglia whilst working at WRA. For example, a history of WRA has acknowledged the role of Bevan-Lewis (Fig. 1, right) in describing spider cells (Todd and Ashworth n.d., p. 224). Another researcher working at WRA around this time, Edwin Goodall (1863–1944; Fig. 1, left), also published on spider cells. The primary research question to be pursued in this paper is how the characterisation and conceptualisation of neuroglial cells in the central nervous system evolved at this single, peripheral, relatively obscure institution over the period of around a decade and a half between 1879 and 1895. A secondary objective is to investigate the research methods of the three individuals, William Bevan-Lewis, Edwin Goodall, and William Lloyd Andriezen, working within a single pathological laboratory, to assess whether their work was solitary or collaborative, incremental or innovative.

## William Bevan-Lewis (1847–1929): spider cells as “scavenger cells”

William Bevan-Lewis came to WRA as an unpaid Clinical Assistant in 1875 and remained there for the rest of his career. He was promoted to Assistant Medical Officer and Pathologist in 1876 and to Medical Superintendent in 1884, and finally retired in 1910 having been President of the Medico-Psychological Association (MPA; forerunner of the Royal College of Psychiatrists) in 1909. Bevan-Lewis was a biological neuropsychiatrist, convinced that mental ailments were morbid conditions of the brain (Hoole 2013). His career was largely devoted to neuropathology, although he did oversee the opening of an outpatient department in 1889, reconstruction of the pathology department to include a complete outfit of laboratories by 1895, and the building of a new acute hospital on the WRA site, opened in 1900. He also witnessed the founding of further branches of WRA, the third and fourth respectively, at Menston in 1888 and Storthes Hall outside Huddersfield in 1904 (Larner and Triarhou 2023). Bevan-Lewis’s work in cortical cytoarchitectonics is perhaps his best remembered research contribution (Lewis and Clarke 1878; Triarhou 2021) but in addition he also made reference to neuroglial elements, specifically to “spider cells”.

The exact date of Bevan-Lewis’s first reference to “spider cells” is uncertain: they were not mentioned in the sequence of five articles on “Methods of preparing, demonstrating, and examining cerebral structure in health and disease” published in *Brain* between 1880 and 1882, papers which became the substance of Bevan-Lewis’s first book, *The Human Brain. Histological and Coarse Methods of Research. A Manual for Students and Asylum Officers* (Bevan-Lewis 1882b). However, reference to “spider-like bodies” appeared in his publication on the cortex cerebri of the sheep, communicated to the Royal Society (by Ferrier) in June 1879 and published the following year:

Immediately beneath the pia mater great numbers of the spider-like bodies known as DEITERS’ cells are met with. They are distributed especially along the course of the blood-vessels which vertically traverse this superficial layer of the cortex. Closely packed beneath the pia mater, their long filaments spread in all directions embracing the nearest blood-vessel. They thin out in numbers below the upper third of this layer, becoming few, and scattered widely apart in the neighbourhood of the small pyramidal layer (Bevan-Lewis 1880, p. 47–48 [capitals in original]).

**Fig. 2:**
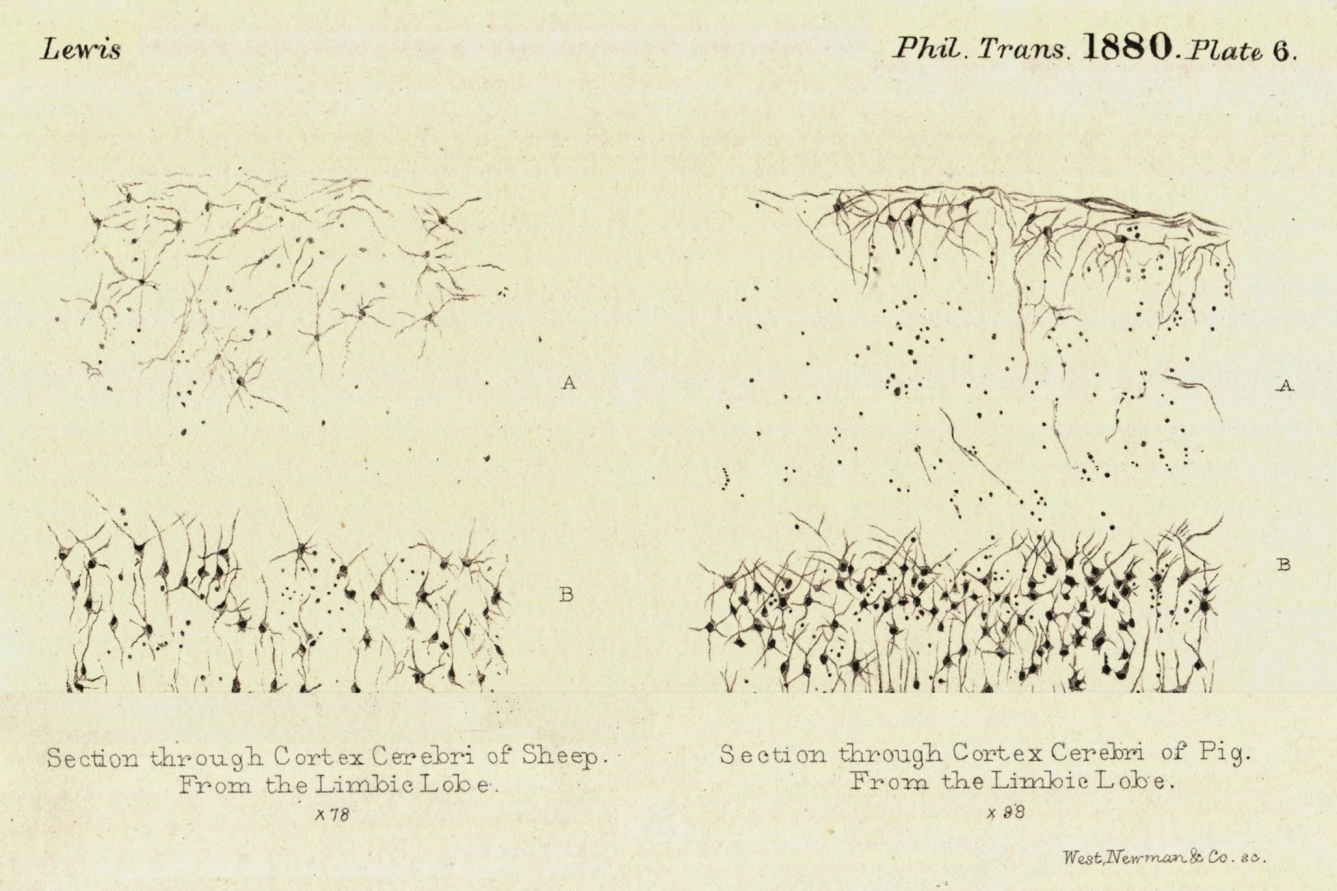
Bevan-Lewis’s illustration of the cerebral cortex of sheep (left) and pig (right) showing “A. First layer exhibiting large numbers of DEITERS’ cells beneath pia mater. B. Second layer of small pyramidal and angular elements”. (From Bevan-Lewis 1880, Plate 6, between pages 59 and 60)

Neither Bevan-Lewis’s illustration of the cerebral cortex (Fig. 2) nor his text was explicit about the tissue staining methods he had used, nor about the microscope makers or models used. However, around the same time, Bevan-Lewis had contributed pathological reports on two cases published by Joseph Plaxton (1846–1904), then Assistant Medical Officer at WRA, in the second of which the “Microscopic Examination of Cerebrum” described “sections stained by logwood, carmine, and aniline” (Plaxton 1878, p. 277).

Spider cells were also described, but not illustrated, in human pathological material, specifically in Bevan-Lewis’s pathological report supplementing a case of ophthalmoplegia externa in conjunction with tabes dorsalis (Buzzard 1882). Describing the microscopical appearances in the spinal cord, probably stained with aniline, Bevan-Lewis stated that:

The spider-like connective cells were far more numerous in the post-commissural than in the posterior radicular zones, and were very scanty or wholly absent in the columns of Goll, where the fine fibrillated intervening connective [sic.] predominated as the morbid element. (Bevan Lewis 1882a, p. 42).

Likewise, in his “Report of microscopical examination of the spinal cord” in the case of “posterior spinal sclerosis” reported by Thomas Buzzard (1831–1919) in *Brain* in January 1884 (Buzzard 1884; Bevan Lewis 1884), when describing the posterior columns of the lumbar region, Bevan-Lewis noted:

Numerous large spider-like connective cells are spread over the field, and much punctated [sic.] fibrous tissue intervenes between the medullated tubuli, which likewise exhibit the segmentation of their medulla as above described. (Bevan Lewis 1884, p. 477).

Despite his illustrations, Deiters had hazarded no explanation as to the function of spider cells beyond the general one of a supporting framework for the nervous tissue proper. This question of function was addressed by Bevan-Lewis in his longest exposition on these cells which appeared in his *Text-book of Mental Diseases*, first published in 1889. In the Preface, he noted that:

In the Pathological Section, I have endeavoured to do justice to certain morbid processes, which appear to me to be of paramount importance in the history of Insanity; and more particularly would I here allude to the functions of the Lymph-connective system of the Brain, and the life-history of the “Scavenger-cell.” (Bevan-Lewis 1889, p. viii).

The contents page listed, as subsections of the chapter on “The Cerebral Cortex”, “The Lymph-connective Elements or Scavenger-cells”, “Vascular Process of Scavenger-cell”, and “Role of Spider- or Scavenger-cell” and, although no such heading appeared in the body of the text, the header of page 85 read “spider-cells of lymph-connective system”. Part of the text reads:

The branched cells which we [Bevan-Lewis] have now described have often been recognised in their morbid modifications, and variously interpreted. Their representatives in healthy brain were first described by Deiters […] but we do not think their true significance has been recognised either as normal or pathological elements of the central nervous system. We incline to regard these elements as comprising the **distal extension of a lymphatic system**, in fact as a **lymph-connective** system permeating the neuroglia in the intervascular area.

The individual elements are excessively delicate and pellucid, their protoplasm appearing almost of fluid consistence, and the vascular process invariably establishing its connection with the lymph sheath of a blood-vessel. In whatever manner these spider cells effect the reabsorption and distribution of the effete material and surplus plasma – whether by direct assimilation into their own structure, and its removal by currents within the protoplasm of the cell and its processes, or by means of a true canalicular system terminating in the lymph sheath – it is an undoubted fact that any arrest to the escape of perivascular lymph from the cortex is immediately followed by a morbid development and hypertrophic condition of this system of **spider cells**, as we shall for the future call these elements of the “lymph-connective system.” (Bevan-Lewis 1889, p. 84 [bold type in original]).

The rationale for ascribing to spider cells a “scavenger” function (although that word did not appear in the text here) was thus their action effecting “the reabsorption and distribution of the effete material and surplus plasma”. That this was one element of the “lymphatic system of the brain” was made explicit by Bevan-Lewis:

The lymphatic system of the brain consists:

1. In the first place, of a distensible lymphatic sheath, loosely applied around the arterioles and venules, containing numerous nucleated cells in its texture—the *adventitial lymph sheath*, the whole being included within a non-distensible channel of the brain-substance, devoid of endothelial lining—the *perivascular channel of His*.
2. In the second place, of a continuation of the cellular elements of this sheath, loosely applied to the **arterio-capillary plexuses**, still contained within a perivascular channel, which now exhibit along the capillary loop sac-like dilatations—the *pericellular sacs*, within which the nerve cell lies, surrounded by plasma.
3. Lastly, of a system of plasmatic cells with numerous prolongations, which are always in intimate connection with the adventitial lymph sheath, and which drain the areas between the vascular branches—these we [Bevan-Lewis] have termed the *lymph-connective elements*. (Bevan-Lewis 1889, p. 85 [italics and bold type in original]).

Bevan-Lewis recognised that these cells may have phagocytic functions:

The term phagocytes, which he [Metschnikoff] employs for those large cells active in the removal of effete material in the frog and other cold-blooded animals, we [Bevan-Lewis] have employed when referring to the spider cell; but we prefer the term scavenger-cell for those fixed tissue-organisms which, as we have seen, have an active physiological and pathological role (Bevan-Lewis 1889, p. 494n).

**Fig. 3:**
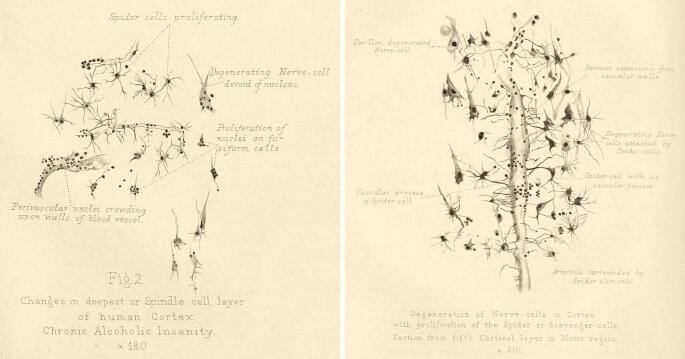
Left, Bevan-Lewis’s illustration of changes in the deepest layer of human cortex in chronic alcoholic insanity: “The blood-vessels which are undergoing fatty degeneration are crowded by perivascular nuclei and surrounded by numerous scavenger-elements, ×180.” (detail from Bevan-Lewis 1889, Plate XVI, Fig. 2, between pages 528 and 529). Right, “Degeneration of Nerve-cells in Human Cortex. Taken from a section of the motor region to illustrate the mode of connection and relationships existing between the scavenger-elements of the lymph-connective system and the cortical blood-vessels. The swollen granular degenerated nerve cells are seen attacked on all hands by the scavenger-elements, ×210.” (detail from Bevan-Lewis 1889, Plate XVIII, between pages 538 and 539)

As hinted in the Preface, more was said on scavenger cells in the Pathological Section of the book, specifically *Pathological Anatomy of General Paralysis* and *Pathology of Chronic Alcoholism*. Once again, the pertinent subsections as listed in the contents did not appear as headings either in the text or as page headers, so all references to scavenger cells were in passing. For example, scavenger cells were noted to be abundant and proliferating in the areas of the spinal cord afflicted in tabes dorsalis (Bevan-Lewis 1889, p. 513, 515) and were ascribed “depurative” functions (Bevan-Lewis 1889, p. 516). In chronic alcoholism, “the abundance of scavenger-cells which pervades the upper or outermost region of the peripheral zone of the cortex lying immediately beneath the pia” was a prominent feature (Bevan-Lewis 1889, p. 528); and in the lower levels of the cortex nerve cells were “undergoing rapid degeneration and removal through the agency of the scavenger-corpuscles, which, as previously explained, act in the capacity of ‘phagocytes,’ and devour the nerve-elements” Bevan-Lewis 1889, p. 532), a process he also illustrated (Fig. 3).

Bevan-Lewis’s classification of scavenger cells was based entirely on inferences drawn from morphological appearances, rather than experimental studies. This question was subsequently addressed by another Pathologist working at WRA.

## Edwin Goodall (1863–1944): “the spider (so-called scavenger) cell of the brain”

Edwin Goodall, a graduate of Guy’s Hospital in London in 1886, worked at WRA in 1890–1891, and again in 1892–1894 as Pathologist and Assistant Medical Officer. He was a prolific writer during this period, publishing on many topics, including clinical and experimental findings, some related to neuropathology and including photography of macroscopic brain pathology. In addition, there were review articles, as well as observations on the effect of “localized inflammatory conditions” or “intercurrent bodily disease” upon mental disorders. In 1895 he was appointed Medical Superintendent of the Joint Counties Asylum, Carmarthen, in Wales, and ten years later at Cardiff City Mental Hospital. He did a stint as editor of the *Journal of Mental Science* (forerunner of the *British Journal of Psychiatry*) and in 1923 was President of the MPA (Anon 1944; Beech 2011). In 1914 he delivered the prestigious Croonian Lectures before the Royal College of Physicians on “the pathology of mental disorders” (Goodall 1914).

Whereas Bevan-Lewis had devoted no specific study or publication to spider cells, Goodall closely examined them – noting the origin of the name with Jastrowitz (Goodall 1894b, p. 394) – and went into press. Whether, as a junior clinician in Bevan-Lewis’s Asylum, this work was undertaken at his senior’s suggestion or request, or whether Goodall hit upon the topic independently is not, to our knowledge, recorded. Certainly, both men had displayed “Microscopic slides, illustrative of recent work on cerebral anatomy and pathology” at a Scottish Meeting of the MPA in Edinburgh in November 1892. According to Goodall’s later entries in the *Medical Directory*, he gained a prize from the MPA for an essay on “The spider cell of the human brain” in 1893, and the first footnote of his publication on the subject stated that it was “Presented to the Medico-Psychological Association, May 1893” (Goodall 1894b, p. 394n1). However, the “Notes and News” section of the *Journal of Mental Science* reporting on the Quarterly MPA meeting of 18th May 1893 made no mention of Goodall, whereas the report on the Annual MPA meeting held on 28th July 1893 stated that “The President […] announced that Dr. [Alfred Walter] Campbell [(1868–1937)], Assistant Medical Officer Rainhill Asylum, Lancashire, had been awarded the bronze medal and prize of ten guineas of the Association […], and the second essay was so good and so near the first that the Council recommended that a prize of five guineas be awarded to Dr. Goodall, of the West Riding Asylum, Wakefield”.

In his subsequent paper, published in the recently established *Journal of Bacteriology and Pathology*, Goodall referred not only to the roles of Virchow and Deiters in elucidating the “connective tissue framework of the brain (neuroglia)” but also to Bevan-Lewis’s proposal for the spider element as “scavenger cell”. The purpose of Goodall’s experimental investigation was thus to inquire into “the nature of the changes exhibited by the spider cells in diseased states of the cerebral cortex” (Goodall 1894b, p. 396).

Using rabbits, he caused brain injury mechanically (brain puncture with sterilised platinum wire) with or without chemical injury (application of cantharidin, turpentine, or carbolic acid). Tissues were examined with the “fresh method” of Bevan-Lewis, and in “a few instances Golgi’s method, as modified by Ramón y Cajal, was employed”. Based on a “Pathological retrospect” he published in the *Journal of Mental Science* in October 1893, Goodall was certainly aware of “A Neuroglia Stain” described by Beneke in Germany (cf. Pollack 1898, p. 148–149). This was “a modification of Weigert’s fibrin-method, by which connective-tissue in the most diverse organs can be consistently stained. Amongst these is the brain; the spider-cells and their prolongations, the fine fibrous networks between pia and cortex and around the ventricles, are stained by the process recommended. The fibrous meshwork of sclerosed tissue is shown remarkably well” (Goodall 1893, p. 580). However, this technique does not appear to have been applied by Goodall in his experimental studies.

Goodall observed enlargement of spider cell bodies following mechanical or chemical brain injury (or both), with increased sensitivity to staining and greater prominence of processes but he did not illustrate these findings, either here or in his book devoted to “The microscopical examination of the human brain” published in the same year (Goodall 1894a). That this cellular hypertrophy occurred without any increase in cell number suggested to Goodall that “abnormal spider cells present in the cerebral cortex in inflammatory states are developed mainly, not from extravasated leucocytes, but out of the fixed connective tissue elements—the pre-existent cells” (Goodall 1894b, p. 400–401). The function of the spider cells was thus deemed “depurative” (the same word once used to describe the function of scavenger cells by Bevan-Lewis 1889, p. 516), the end effect being the formation of sclerosed tissue, as had been previously suggested by Bevan-Lewis. However, although Goodall’s study was attempting to “see if any evidence of the phagocytic action of spider upon nerve cell could be obtained” he was obliged to admit that “It must be stated that none was forthcoming” (Goodall 1894b, p. 404).

The exact date of Goodall’s studies on spider cells in the period 1892–1894 is not known but it transpires that, as noted in a footnote to Goodall’s paper (Goodall 1894b, p. 403n1), another pathologist based at WRA was also working on “spider cells” around this time: William Lloyd Andriezen.

## W. Lloyd Andriezen (c. 1870–1906): dividing the category of “spider cells”

William Lloyd Andriezen arrived at WRA in January 1893 as Pathologist and Assistant Medical Officer having previously trained at University College London (UCL). Whilst at UCL he had already commenced comparative and zoological work in pathology, for example on the histology of the pituitary gland and cerebral cortex as well as on neuroglia, and some of this material came to publication during his time at WRA (Larner 2025). Availing himself of the Golgi staining technique with silver chromate, Andriezen’s key papers on neuroglia appeared in 1893 (Andriezen 1893a, b, 1894a), firstly in the *British Medical Journal* issue of 29th July 1893.

**Fig. 4:**
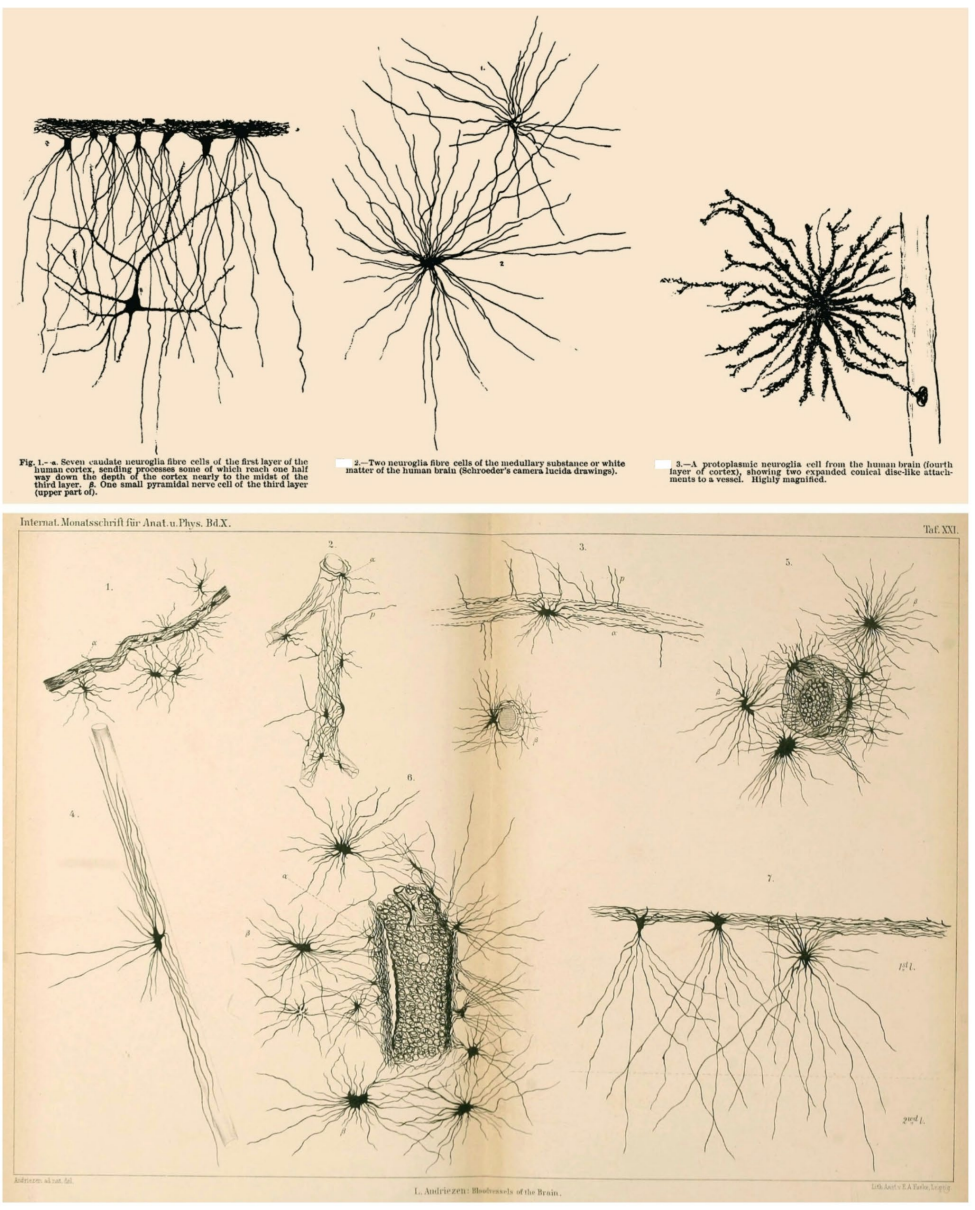
Andriezen’s drawings of various forms of glial cells, neuroglial fibre cell and protoplasmic neuroglial cell, Golgi silver chromate stain. (Andriezen 1893b; top; Andriezen 1893a; below)

As the Golgi staining technique, a variant of which Andriezen had developed (Andriezen 1893a, 1894a), gave more detailed images of cells and their ramifications, he was able to subdivide neuroglia into different types. Based initially on morphological grounds, Andriezen distinguished the “neuroglia fibre cell” (Andriezen 1893b) or “fibre elements” (Andriezen 1893a) from “the protoplasmic neuroglia cell” (Andriezen 1893b) or “protoplasmic cell elements” (Andriezen 1893a). The latter were found to be abundant in grey matter and formed a perivascular feltwork supporting these elements and protecting nerve cells from damage, whereas the fibrous forms were found especially in the white matter (Fig. 4).

In his “Conjectures on the anatomical mechanism of ideation, association and attention”, Ramón y Cajal (1895a, b) remarked that diverse phases of neuroglial cells of the grey matter were undoubtedly observed by Andriezen 1893a, b; Retzius (1894), and others, although they were believed to be mere morphological varieties of the typical cell of Deiters. According to Andriezen 1893a, b; Retzius (1894), and Ramón y Cajal (1895a, b), neuroglial cells of the grey matter exhibit a particular and highly typical physiognomy. Ramón y Cajal further made a distinction between astroglia located in the white or the grey matter, respectively, fibrous and protoplasmic astrocytes, an old histological classification (Andriezen 1893b; Weigert 1895) that still holds today (Peters et al. 1991, p. 277–284).

Andriezen contrasted his subdivision of neuroglia with then existing views: “Hitherto all neuroglia cells in the adult brains have been included under one category of ‘spider’ cells (Deiters, Meynert, and others)” (Andriezen 1893b, p. 228). No mention of “scavenger” cells or phagocytosis was made in either of his key papers (Andriezen 1893a, b).

In later papers, Andriezen examined cases of “alcoholic insanity” and “epileptic idiocy and epileptic imbecility” (Andriezen 1894b, 1897) and reported changes in the neuroglia. In the former, he noted softening and swelling in the neuroglial elements and the formation of “colloid bodies” originating from the “fibre cells” (Andriezen 1894b, p. 684–685) but did not provide illustrations. In the latter – his affiliation now recorded as “late WRA Pathologist”, although some of the material had been presented to the Leeds and West Riding Medico-Chirurgical Society in April 1895 (Andriezen 1895) – he noted not only histopathological alterations in neurones but also reported changes of a “striking nature” in the neuroglial cells. Specifically:

There is a sclerotic overgrowth of the neuroglia fibre cells (diffuse or focal, and in the latter case often corresponding to a particular vascular territory), with a corresponding slow irritation, destruction, and final atrophy of the nerve cells and fibres in the same area.

[…] diffuse strands and sheets of sclerosed tissue are seen, in some places concentrated into firm almost fibroid islets; in other places finely diffused throughout, and infiltrating the brain substance. Where these changes occur in the deeper cortical and neighbouring parts, the naked eye difference of colour between cortex and white substance is often lost, and the tissue so infiltrated feels firm, and even hard. Where several such spots or patches are aggregated the brain substance feels knotty when pressed. (Andriezen 1897, p. 1082).

This “sclerosis”, presumably what we might now term gliosis or astrocytosis, was not illustrated by Andriezen. A later WRA pathologist, Francis O. Simpson, refuted Andriezen’s claims as to the frequency with which sclerosis was seen in epileptic “idiocy and imbecility” (degrees of intellectual impairment in current terms), questioning the diagnosis of some of Andriezen’s cases based on his retrospective examination of the WRA postmortem books (Simpson 1900, p. 39, 40).

## Discussion

Whilst Andriezen’s work has occasionally been mentioned in histories of glia (e.g. Glees 1955, p. 70; Somjen 1988; Kettenmann and Verkhratsky 2008; Fan and Agid 2018; Brenner and Parpura 2024), neither he nor Bevan-Lewis nor Goodall are mentioned in a more recent (otherwise comprehensive) account by Kettenmann et al. (2025), although the supplement prepared by these authors containing reference lists of early workers in the field included both Bevan-Lewis (1889) and Andriezen (1893a). Goodall’s work (1894b) seems to have been entirely ignored by posterity. It is of note that one of Andriezen’s key publications (Andriezen 1893a) appeared in a German journal, albeit written in English; as German was recognised to be the lingua franca of research at that time (Kettenmann et al. 2025, p. 891), Andriezen was presumably hoping to widen the audience for his work. The paucity of recognition of each of these three investigators in the history of neuroglial cells justifies the current re-examination of their contributions.

As Bevan-Lewis had been Medical Superintendent at WRA since 1884, he must have been instrumental in the appointments of both Goodall and Andriezen. All three were at WRA in the years 1893 to 1895, and hence presumably worked in the same laboratory and possibly at the same bench. Hence it is pertinent to ask to what extent they were aware of one another’s work, whether they influenced one another, and whether there was any collaborative effort. The documentary evidence is largely negative, with no positive evidence to suggest any collaboration.

Bevan-Lewis had been longest in the field, a recognised expert in cerebral histology. He had long observed spider cells and postulated a scavenger function for them, an innovative view which he held persistently: the second, 1899, edition of his *Text-book of Mental Diseases* has identical wording in the passages quoted above from the first edition of 1889, along with a new footnote containing “references to some few of the articles bearing directly upon phagocytosis”, including Goodall’s paper (Bevan-Lewis 1899, p. 99–101).

That Goodall set out explicitly to test experimentally Bevan-Lewis’s claims of a scavenger function for spider cells seems clear from his paper, and Bevan-Lewis may have been aware of this, based on his reference to Goodall (1894b) in the second edition of the *Text-book of Mental Diseases*.

In contrast, Andriezen seems to have worked alone, perhaps because his studies had commenced elsewhere (UCL) and because he had developed expertise in the use of the Golgi technique which seems to have been used sparingly by Goodall and little, if at all, by Bevan-Lewis (e.g. Bevan-Lewis 1899, p. 65, 78, 81). Andriezen referenced neither Bevan-Lewis nor Goodall; it is questionable what, if any, influence Bevan-Lewis, as his ultimate boss, had on Andriezen. Indeed, in contrasting his subdivision of neuroglia with then existing views, Andriezen had noted that:

Hitherto all neuroglia cells in the adult brains have been included under one category of ‘spider’ cells (Deiters, Meynert, and others). (Andriezen 1893b, p. 228).

It is difficult not to see Bevan-Lewis as one of these “others”, and possibly also Goodall. Whilst Goodall explicitly stated, having seen Andriezen’s *BMJ* paper (Andriezen 1893b), that his work was independent of Andriezen (Goodall 1894b, p. 403n1), the latter’s work was not referenced in the second edition of Bevan-Lewis’s *Text-book*. Whether this omission was mere oversight or a wilful exclusion on Bevan-Lewis’s part cannot be decided with the currently available evidence, but it appears to be in keeping with his previous non-citation of the work of his colleague and predecessor, Herbert Major, on cortical cytoarchitectonics when he came to publish on the subject (Lewis and Clarke 1878; Larner and Triarhou 2024). Perhaps he felt Andriezen had added little to his conceptions of spider cells and their scavenger function.

In sum, these observations would seem to endorse one view of posterity that dialogue between researchers at WRA was lacking in this period (Wallis 2017, p. 127), in contrast to the situation when Crichton-Browne was Superintendent (1866 to 1876) and the pathological laboratory had first been established. It remains an open question whether greater progress might have been made had Bevan-Lewis, Goodall, and Andriezen developed some form of collaboration. Ultimately, Andriezen’s early death at the age of 36 would have precluded any longer-term association.

Aside from these local issues, what general impact did these studies emanating from WRA have, if any? Unlike the situation in the 1870s when the work pursued in the WRA pathological laboratory was of both national and international interest – for example through its links with Ferrier and its own house journal, the *West Riding Lunatic Asylum Medical Reports* (*WRLAMR*) – by the 1890s it had become relatively obscure. *WRLAMR* had ceased publication in 1876 and the last of Bevan-Lewis four papers presented at the Royal Society (all were communicated by Ferrier) dated to 1881. Thus little note seems to have been taken of the work of Bevan-Lewis and Goodall, and leading authors of this era continued to overlook the broader physiological significance of neuroglial cells; for example, Campbell (1905, p. 10) was of the view that they “can be dismissed without further remark”. The possible phagocytic (“scavenger”) function of spider cells did not gain general acceptance until the description of microglia (“the third element”) in 1919 by Cajal’s Spanish pupil Pío del Río-Hortega (1882–1945) (Triarhou 2025).

Andriezen’s subdivision of spider cells into different forms coincided with a similar report by the Swiss anatomist Albert von Kölliker (1817–1905) who described *Kurzstrahler* (“short beamer”) and *Langstrahler* (“long beamer”) glial cells. Some historians have thus been of the view that von Kölliker was “first to distinguish two variants of glial cells” (Kettermann 2025, p 899), entirely ignoring Andriezen, whereas others have jointly ascribed this discovery (e.g. Brenner and Parpura 2024). Both Andriezen and von Kölliker were apparently unaware of Golgi’s (1871, 1872, 1885) differentiation of neuroglial cell forms. Andriezen’s observations were the stimulus for Carl Ludwig Schleich’s (1859–1922) 1894 dynamic view of glia as active rather than merely passive static elements in the brain (Dierig 1994).

The diversity of neuroglial cells had thus been recognised, and shortly thereafter, from 1894, Gustaf Retzius (1842–1919) was describing other forms. Again it was not until the work of del Río-Hortega that oligodendrocytes, as well as microglia, became established within the class of neuroglial cells.

## Data Availability

No datasets were generated or analysed during the current study.
